# Comparison of postoperative pain in children after maintenance anaesthesia with propofol or sevoflurane: a systematic review and meta-analysis

**DOI:** 10.1016/j.bja.2024.03.022

**Published:** 2024-04-26

**Authors:** Bushra M. Abdallah, Amgad M. Elshoeibi, Nouran ElTantawi, Mariah Arif, Razan F. Hourani, Aishat F. Akomolafe, Mahmoud N. Hamwi, Fathima R. Mahmood, Kemal T. Saracoglu, Ayten Saracoglu, Tawanda Chivese

**Affiliations:** 1 College of Medicine, QU Health, Qatar University, Doha, Qatar; 2 Department of Anaesthesiology, ICU, and Perioperative Medicine, Hazm Mebaireek General Hospital, Hamad Medical Corporation, Doha, Qatar; 3 Department of Anaesthesiology, ICU, and Perioperative Medicine, Aisha Bint Hamad Al-Attiyah Hospital, Hamad Medical Corporation, Doha, Qatar

**Keywords:** children, maintenance anaesthesia, postoperative pain, propofol, sevoflurane

## Abstract

**Background:**

Propofol and sevoflurane are two of the most commonly used anaesthetics for paediatric surgery. Data from some clinical trials suggest that postoperative pain incidence is lower when propofol is used for maintenance of anaesthesia compared with sevoflurane, although this is not clear.

**Methods:**

This meta-analysis compared postoperative pain following maintenance of anaesthesia with propofol or sevoflurane in paediatric surgeries. PubMed Medline, Embase, Scopus, Web of Science and Cochrane Library were searched for randomised controlled trials (RCTs) that compared postoperative pain between sevoflurane and propofol anaesthesia in children. After quality assessment, a meta-analysis was carried out using bias-adjusted inverse heterogeneity methods, heterogeneity using *I*^2^ and publication bias using Doi plots.

**Results:**

In total, 13 RCTs with 1174 children were included. The overall synthesis suggested nearly two-fold higher odds of overall postoperative pain in the sevoflurane group compared with the propofol group (odds ratio [OR] 1.88, 95% confidence interval [CI] 1.12–3.15, *I*^2^=58.2%). Further, children in the sevoflurane group had higher odds of having higher pain scores (OR 3.18, 95% CI 1.83–5.53, *I*^2^=20.9%), and a 60% increase in the odds of requiring postoperative rescue analgesia compared with propofol (OR 1.60, 95% CI 0.89–2.88, *I*^2^=58.2%).

**Conclusions:**

Children maintained on inhalational sevoflurane had higher odds of postoperative pain compared with those maintained on propofol. The results also suggest that sevoflurane is associated with higher odds of needing postoperative rescue analgesia compared with propofol.

**Registration:**

The protocol for this systematic review and meta-analysis was registered on the International Prospective Register of Systematic Reviews (PROSPERO) with registration ID CRD42023445913.

**Editor's key points**

• Evidence of the impact on postoperative pain of the use of sevoflurane and propofol for the maintenance of general anaesthesia is inconclusive.
• This meta-analysis of 13 RCTs suggests that use of sevoflurane is associated with a larger risk of postoperative pain in children in comparison with use of propofol.
• Further research is required to confirm this finding and to explore the mechanisms involved.

Postoperative pain remains a key problem, especially in paediatric populations. Regardless of the type of analgesic treatment provided, the proportion of children who report moderate to severe postoperative pain remains significant.^1^, ^2^, ^3^ This has led to investigations on the type of anaesthesia and their possible effects on postoperative pain.

Propofol is frequently used for total intravenous anaesthesia (TIVA) or after inhalation induction with volatile anaesthetics. In children, propofol has a higher volume of distribution, has a shorter elimination half-life, and is cleared from the body more quickly than in adults.^4^ Thus, although a similar blood concentration of propofol is needed for effective anaesthesia in both children and adults, the dose needed for infusion of propofol in children is around two times that of adults.^5^^,^^6^ Propofol is commonly chosen as the sedative–hypnotic agent for maintaining general anaesthesia. It is typically administered as a continuous infusion using a syringe pump or smart pump. Factors such as older age, hypovolaemia, vasodilation, myocardial dysfunction, and coadministration of other agents can require dose reduction.^7^, ^8^, ^9^

Some advantages of propofol are rapid onset^10^ and recovery,^11^ in addition to its antiemetic, anticonvulsive, antipruritic, and bronchodilatory properties.^12^^,^^13^ Moreover, it is suitable for patients with renal or hepatic insufficiency.^14^ In addition, propofol used in TIVA can have antioxidant, anti-inflammatory, and immunomodulatory effects.^12^ Clinically significant adverse effects of propofol are minimal when titrated to the desired depth of anaesthesia. However, hypotension can occur at higher doses in susceptible patients as a result of venous and arterial dilation. Respiratory depression is a known side-effect, which is dose dependent.^15^

Apart from the aforementioned advantages, some data, though inconclusive, suggest that propofol can result in less postoperative pain compared with the alternative inhalation sevoflurane anaesthesia. Some randomised controlled trials (RCTs) have shown that maintenance anaesthesia using propofol is associated with less postoperative pain.^5^^,^^6^ However, other RCTs found no difference between propofol and sevoflurane in postoperative pain occurrence and intensity.^16^^,^^17^ With the exception of a meta-analysis that examined the safety of the two agents for general anaesthesia in children,^18^ no other meta-analysis has been conducted regarding this topic. In the aforementioned meta-analysis, where postoperative pain associated with the two agents was investigated as a secondary outcome, the findings lacked certainty because of the small number of included studies. The current meta-analysis addresses some of these shortcomings and includes more than two times the number of RCTs that were in the previous meta-analysis. Therefore, this meta-analysis assessed the effect of maintenance anaesthesia with propofol compared with sevoflurane on postoperative pain in children.

## Methods

### Study design and protocol registration

This is a systematic review and meta-analysis of RCTs. It adheres to the Preferred Reporting Items for Systematic Reviews and Meta-Analyses (PRISMA) 2020 guidelines (Supplementary Table S1).^19^ The protocol for this systematic review and meta-analysis was registered on the International Prospective Register of Systematic Reviews (PROSPERO) with registration ID CRD42023445913.^20^

### Data sources

We searched PubMed Medline, Embase, Scopus, Web of Science, and Cochrane Library with no language or date restrictions. We also screened the references of included studies for additional studies. Authors were contacted directly if full text reports were not found.

### Search methods

The search strategy was developed using the population, *i*ntervention, *c*omparator, *o*utcome (PICO) question, ‘In children undergoing surgical operations, is maintenance anaesthesia using propofol compared with sevoflurane associated with more or less postoperative pain?‘. The PICO terms, children, propofol, sevoflurane, and postoperative pain, were then used as search terms. For each term, we used both keywords and Medical Subject Headings (MeSH) terms in PubMed, while only keywords were used in the other databases. The MeSH terms used were ‘Pain, Postoperative’, ‘Sevoflurane’, and ‘Propofol’, in addition to keyword terms such as ‘Propofol’, ‘Sevoflurane’, ‘Postoperative pain’, ‘rescue analgesia’, ‘children’, ‘pediatric’, and their synonyms. During the initial search, we did not impose any restrictions on language or publication date. To extend our search to other databases, we used the Polyglot translator to adapt our search strategy for Embase, Scopus, Web of Science, and the Cochrane Library.^21^ The complete search strategy for each database can be found in Supplementary Tables S2–S6.

### Procedure for selection of studies

The citations that were identified from the searches were transferred to EndNote 20 for duplicate removal before being uploaded to the Rayyan Systematic Review Management platform (https://www.rayyan.ai/) for preliminary screening based on their titles and abstracts.^22^ Two pairs of independent investigators (*RFH & AFA and MNH & FRM*) manually assessed the title and abstract of the retrieved articles for eligibility. In case of disagreement between the two investigators in a pair, a third investigator (*BMA*) made the final decision. Abstracts available in languages other than English were translated using Google Translate and then screened for eligibility. The study records identified from the titles and abstracts were retrieved and underwent full-text screening by (*BMA & AME and NET & MA*).

### Eligibility criteria

Two pairs of independent investigators screened the full text of potentially relevant articles for eligibility. RCTs comparing the postoperative pain after administration of maintenance anaesthesia with propofol or sevoflurane to children undergoing surgeries were included. Narrative reviews, quasi-experimental studies, observational studies, letters, opinions, and other non-original articles were excluded. Studies without primary data and studies that did not include paediatric populations or did not measure postoperative pain adequately were also excluded.

### Outcomes

The primary outcome was overall postoperative pain assessed as the need for rescue analgesia. If a study did not report this outcome, we extracted pain assessed from a cutoff using a pain assessment tool. The two secondary outcomes were; (1) pain scores from pain assessment tools only and (2) the requirement of rescue analgesia only.

### Data extraction

The following characteristics were extracted from each study: authors, year of publication, country in which the study was performed, and the study period. Furthermore, we gathered data regarding the type of surgical procedure performed, sample size in each group, and their patient characteristics, including age, gender, and American Society of Anesthesiologists (ASA) physical status. We also extracted information on the type, dosage, and form of induction/maintenance anaesthetic used in each group and the mean duration of anaesthesia. To evaluate postoperative pain, we extracted information about the type of pain score used to assess postoperative pain, and the mean score within each group at different times. We also captured data on the numbers of participants with pain based on score cutoffs, and the maximum pain score recorded. To evaluate postoperative pain management, we extracted information about the number of participants who required rescue analgesia within each group.

### Assessment of the quality of included studies

The assessment of the quality of included studies was conducted by two pairs of independent investigators (*BMA & AME and NET* and *MA*) using the Methodological Standard for Epidemiological Research (MASTER) scale, which comprises 36 safeguards under seven methodological standards.^23^ Any disagreements between the two authors were resolved through discussion. The seven standards of the MASTER scale are as follows: equal recruitment (items 1–4), equal retention (items 5–9), equal ascertainment (items 10–16), equal implementation (items 17–22), equal prognosis (items 23–28), sufficient analysis (items 29–31), and temporal precedence (items 32–36).^23^

### Synthesis of findings

Data that could not be synthesised in a meta-analysis were presented in tables and analysed descriptively in the text. The quality effects model was used for bias-adjusted synthesis of the outcome estimates. This model uses an inverse variance heterogeneity meta-analysis synthesis and assumes that the effects from different studies are estimating a common effect. The model compensates for variability arising from differences in methodological quality by redistributing study weights based on quality ranking, thereby adjusting for bias in synthesis.^24^^,^^25^ The results of the quality assessment were used to compute relative rankings.^26^ To enable comparisons with other studies, estimates from the random effects model were also generated. Outcome estimates and their pooled values from both models were depicted using forest plots. To assess heterogeneity, we used the *I*^2^ statistic and the Cochrane *Q* test *P*-value. Statistically significant heterogeneity was identified when the Cochrane *Q* test yielded *P*<0.05 or when *I*^2^ exceeded 50%.^27^ Doi plots, the Luis Furuya-Kanamori (LFK) index,^28^ funnel plots, and Egger's regression test *P*-value (significance at <0.1)^29^ were used to assess publication bias. We also performed a leave-one-out sensitivity analysis by systematically removing each study and examining how this affected the meta-analysis estimates. The Grading of Recommendations, Assessment, Development, and Evaluations (GRADE) framework was used to assess the certainty of the evidence.^30^^,^^31^ All analyses were conducted using Stata 17.0 (StataCorp, College Station, TX, USA).

Subgroup analysis was performed for the type of surgery and intraoperative analgesic management. The subgroups for the type of surgery were based on the anatomical site of the surgery and included dental surgery, otolaryngology, general surgery, ophthalmology, orthopaedics, urology, and multiple surgery types. The four subgroups of intraoperative analgesic management were IV opioids alone, neuraxial alone, neuraxial and IV opioids, and studies where intraoperative pain management was not reported.

### Ethics approval

This review used secondary data from peer-reviewed published studies and does not require ethical clearance.

## Results

### Search output

**Fig 1:**
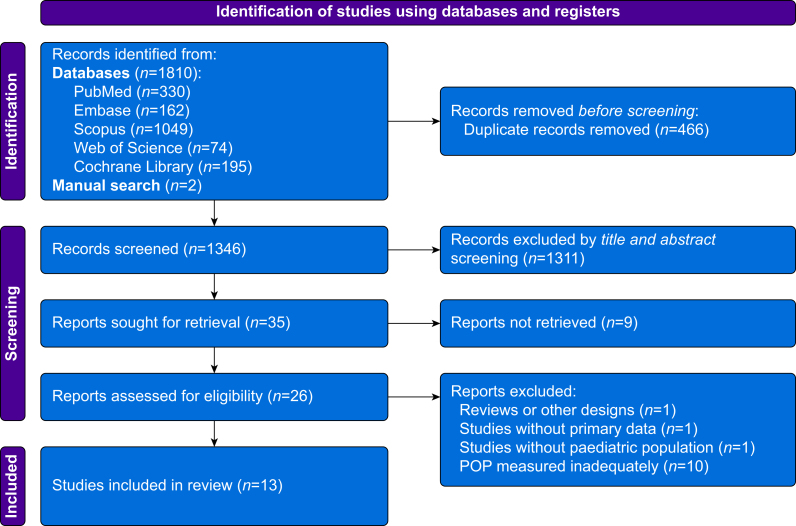
PRISMA flowchart. POP, postoperative pain.

Figure 1 provides an overview of the search process. A total of 1810 records were identified from electronic searches, and two additional records were identified through manual search. Using EndNote (Clarivate, London, UK), 466 duplicates were removed. The remaining 1346 study records were imported into Rayyan (Rayyan Systems, Inc., Cambridge, MA, USA), where a total of 1311 records were excluded through title and abstract screening. Nine of the 35 remaining study reports were not available, thus leaving 26 studies for full-text screening. Thirteen studies were excluded for the reasons listed in the PRISMA Flowchart (Fig. 1) and in Supplementary Table S7, leaving 13 studies which were included.

### Characteristics of included studies

In total, 13 RCTs with 1174 patients (594 on sevoflurane and 580 on propofol) were included in the meta-analysis. Participants were aged between 2 months and 16 yr. All the participants in the studies documenting ASA physical status had status 1 and 2. The types of surgery of the 13 RCTs mainly included hernia repair, cleft lip and palate repair, adenotonsillectomy, strabismus surgery, and dental surgery. The main characteristics of the 13 studies are summarised in Table 1.

**Table 1:** Characteristics of the included papers. ASA, American Society of Anesthesiologists; FLACC, Face, Legs, Activity, Cry, and Consolability; FPS, Faces Pain Scale; NR, not reported; VRS, Verbal Rating Scale; VAS, Visual Analogue Scale.

Study	Year	Country	Number of participants	Age	Type of surgery	ASA class	Maintenance anaesthesia dose		Type of pain scale
							Sevoflurane	Propofol	
Guard and colleagues^32^	1998	Canada	50	2–8 yr	Penile, hernia/hydrocele, orchidopexy, hypospadias and hernia	NR	2.5%	5–10 mg kg^−1^ h^−1^	NR
Rüsch and colleagues^33^	1999	Germany	105	3–8 yr	Strabismus repair	NR	1%–1.5%	10 mg kg^−1^ h^−1^	NR
Lovstad and Stoen^34^	2001	Norway	42	NR	Osteotomy	NR	NR	4–10 mg kg^−1^ h^−1^	5-point categorical VRS
Schmidt and colleagues^35^	2001	Germany	120	6 months to 16 yr	Lower abdominal surgery	1 or 2	2.4%–3.3%	7.5 mg kg^−1^ h^−1^	Modified Objective Pain Discomfort Scale
Cohen and colleagues^36^	2003	USA	53	2 months–36 months	General surgery, urology, otolaryngology, orthopaedics, plastic surgery, ophthalmology	NR	1.5%–2.5%	200 μg kg^−1^ min^−1^	Objective Pain Scale
Cohen and colleagues^37^	2004	USA	56	<3 yr	Infraumbilical and suprasternal procedures	NR	1.5%–2.5%	200 μg kg^−1^ min^−1^	NR
Auerswald and colleagues^38^	2006	Germany	103	1–5 yr	Adenoidectomy and adenotonsillectomy	NR	2%–3%	5 mg kg^−1^ h^−1^	Smiley–Wert
Chandler and colleagues^39^	2013	Canada	94	2–6 yr	Strabismus repair	1 or 2	NR	NR	FLACC
Hasani and colleagues^3^	2013	Kosova	88	3–6 yr	Hernia repair	1 or 2	1.5%–2%	9 mg kg^−1^ h^−1^	FPS
Oriby and Elrashidy^40^	2021	Qatar	84	3–11 yr	Strabismus repair	1 or 2	NR	4 mg kg^−1^ h^−1^	FLACC
Sheikhzade and colleagues^41^	2021	Iran	80	2–10 yr	Herniotomy, orchiopexy, frenulectomy, and sigmoidoscopy	1 or 2	2%–3%	100–250 μg kg^−1^ min^−1^	Wong–Baker Faces Pain Rating Scale
Lopéz and colleagues^42^	1999	NR	120	6 months to 12 yr	Minor surgery below the umbilicus (e.g. inguinal hernia, circumcision, orchidopexy)	NR	1.7%	5 mg kg^−1^ h^−1^	NR
König and colleagues^43^	2009	USA	179	2–12 yr	Ambulatory dental surgery	NR	2%	120–250 μg kg^−1^ min^−1^	FLACC, Oucher, and VAS

### Assessment of the quality of included studies

The included studies were of generally good quality with MASTER scale scores ranging from 28 to 33, and an average of 31 out of 36. The higher the score, the higher the quality a study had, that is, the study would have fulfilled a greater number of safeguards against systematic error. Most studies had safeguards present in five of the seven domains that the MASTER scale assesses, that is, equal recruitment, equal retention, implementation, sufficient analysis, and good temporal precedence. However, there were deficiencies in safeguards for equal ascertainment and equal prognosis in some studies. The individual assessments for all the studies are shown in Supplementary Table S8.

### Primary outcome

**Fig 2:**
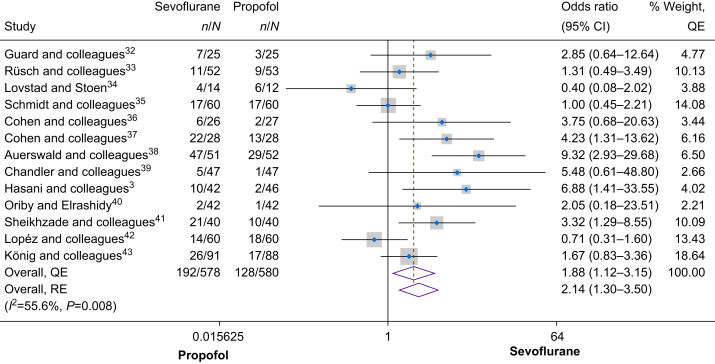
Forest plot of the primary outcome (overall postoperative pain). See also.^3^^,^^32^, ^33^, ^34^, ^35^, ^36^, ^37^, ^38^, ^39^, ^40^, ^41^, ^42^, ^43^ CI, confidence interval; QE, quality effects; RE, random effects.

Thirteen RCTs, which included 1158 children, examined the incidence of overall postoperative pain, measured through pain assessment tools or the requirement for rescue analgesia. As shown in Figure 2, a meta-analysis of the trials showed higher odds of overall postoperative pain in the sevoflurane group compared with the propofol group (odds ratio [OR] 1.88, 95% confidence interval [CI] 1.12–3.15). There was moderate heterogeneity between the studies (*I*^2^=55.6%, Cochrane's *Q*=0.008). A leave-one-out-analysis showed that Lopéz^42^ had the greatest influence, although leaving this study out did not alter the conclusions of the meta-analysis (Supplementary Fig. S1). Assessment of publication bias showed minor positive asymmetry (Doi plot in Supplementary Fig. S2, LFK=1.66, Funnel plot in Supplementary Fig. S3, Egger's *P*=0.182), suggesting that there were no significant concerns for publication bias. Similar findings were obtained when using the random effects model, showing that use of sevoflurane was associated with a higher overall postoperative pain (OR 2.14, 95% CI 1.30–3.50).

### Secondary outcomes

#### Postoperative pain measured using pain assessment tools only

**Fig 3:**
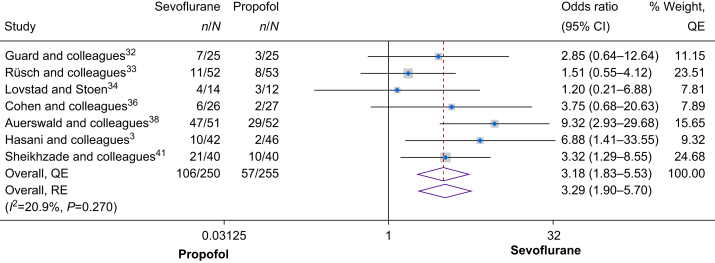
Forest plot of the secondary outcome (postoperative pain measured using pain assessment tools only). See also.^3^^,^^32^, ^33^, ^34^^,^^36^^,^^38^^,^^41^ CI, confidence interval; QE, quality effects; RE, random effects.

Postoperative pain measured using pain assessment tools only was assessed by seven studies with a total of 505 patients. The meta-analysis estimate of these studies showed that use of sevoflurane for maintenance anaesthesia was associated with a significantly higher odds of having postoperative pain and higher pain scores (OR 3.18, 95% CI 1.83–5.53, *I*^2^=20.9%, Cochrane's *Q*=0.270; Fig. 3). Assessment of publication bias showed no asymmetry (Doi Plot in Supplementary Fig. S4, LFK=–0.13, Funnel plot in Supplementary Fig. S5, Egger's *P*=0.895). Similar findings were obtained when the random effects model was utilised (OR 3.29, 95% CI 1.90–5.70).

#### Requirement of rescue analgesia

**Fig 4:**
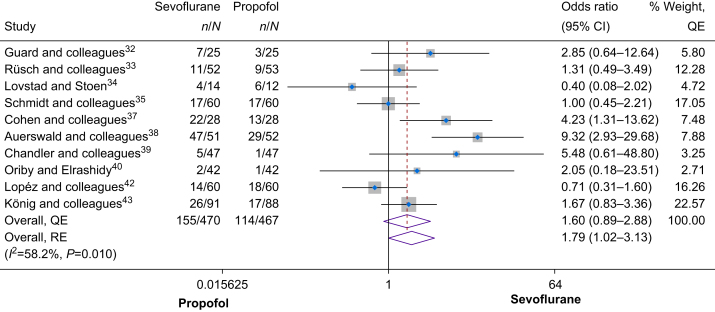
Forest plot of the secondary outcome (requirement of rescue analgesia). See also.^32^, ^33^, ^34^, ^35^^,^^37^, ^38^, ^39^, ^40^^,^^42^^,^^43^ CI, confidence interval; QE, quality effects; RE, random effects.

Postoperative rescue analgesia requirement was investigated in 10 studies involving a total of 937 patients. In the overall synthesis (Fig. 4), use of sevoflurane for maintenance anaesthesia was associated with a 60% increase in odds of requiring postoperative rescue analgesia compared with propofol (OR 1.60, 95% CI 0.89–2.88), with moderate heterogeneity among the studies (*I*^2^=58.2%, Cochrane's *Q*=0.01). Sensitivity analysis (leave-one-out) showed that the results were robust, consistently showing that use of sevoflurane was associated with increased odds of requiring postoperative rescue analgesia (Supplementary Fig. S6). The results remained similar when using the random effects model (OR 1.79, 95% CI 1.02–3.13). Assessment of publication bias indicated minor positive asymmetry (Doi plot in Supplementary Fig. S7, LFK=1.68, funnel plot in Supplementary Fig. S8, Egger's *P*=0.363), suggesting no significant concerns for publication bias.

### Subgroup analysis

For subgroup analysis by type of surgery, the findings from all the subgroups, except for orthopaedics and urology, were consistent with the overall analysis, showing increased odds of overall postoperative pain with sevoflurane compared to propofol (Supplementary Fig. S9). The orthopaedic subgroup, which included only one RCT^34^ suggested that sevoflurane was associated with decreased odds of overall postoperative pain compared with propofol (OR 0.40, 95% CI 0.08–2.02), although caution should be exercised in the interpretation of such findings from subgroups with few studies. In contrast, the urology subgroup, which included two studies,^32^^,^^42^ suggested no difference in overall postoperative pain with both sevoflurane and propofol (OR 1.01, 95% CI 0.25–4.05).

Subgroup analysis by intraoperative analgesic management showed that the RCTs that did not report the use of intraoperative analgesia had higher odds of overall postoperative pain in the sevoflurane group compared with the propofol group (OR 3.22, 95% CI 1.46–7.12). Similar results were found in the neuraxial and i.v. opioids subgroup, which included two trials (OR 4.06, 95% CI 1.55–10.66). In contrast, no difference in the odds of overall postoperative pain was found in the IV opioids alone subgroup and the neuraxial analgesia alone subgroup (Supplementary Fig. S10).

### GRADE rating for the primary outcome

The GRADE rating for the primary outcome (overall postoperative pain) was downgraded one level because of inconsistency as the heterogeneity was moderately high. Hence, the GRADE rating for the primary outcome that intraoperative maintenance with sevoflurane leads to higher overall postoperative pain compared with propofol in children was of moderate certainty.

## Discussion

Using data from 13 RCTs, this meta-analysis showed a higher likelihood of experiencing postoperative pain when sevoflurane was used compared with propofol anaesthesia in children. Similar results were observed for the secondary outcomes of pain scores using pain assessment tools and the need for rescue analgesia.

We found that use of sevoflurane increased the odds of postoperative pain by almost two-fold, although significant heterogeneity was noted. Several different pain assessment tools were used across the RCTs, including the Faces Pain Scale; Visual Analogue Scale; Objective Pain Scale; and Face, Legs, Activity, Cry, and Consolability Scale, which could also explain the increased heterogeneity in the analysis of the primary outcome. Our findings are consistent with findings from one meta-analysis which compared the two agents in adults and found that propofol was associated with a lower postoperative pain intensity.^44^ There are no other meta-analyses which have compared postoperative pain between sevoflurane and propofol as a primary outcome in children. However, one meta-analysis analysed postoperative pain as a secondary outcome and showed increased odds of having postoperative pain in the sevoflurane group compared with the propofol group in an analysis of six trials (OR 1.72, 95% CI 1.11–2.64).^18^ Despite including fewer RCTs in their analysis, their findings were consistent with ours. Our findings conclusively add to this evidence and suggest a need to monitor pain and consider planning for better pain management in children undergoing sevoflurane anaesthesia.

We found a three-fold increase in the odds of experiencing postoperative pain on a pain assessment scale (OR 3.18, 95% CI 1.83–5.53) for children maintained on sevoflurane compared with those on propofol. Overall, these findings strongly suggest that using sevoflurane for maintenance anaesthesia is consistently associated with a higher likelihood of postoperative pain measured by pain assessment tools when compared with the use of propofol. These findings are similar to those obtained in the meta-analysis by Peng and colleagues^44^ in adults maintained on propofol *vs* inhalational anaesthesia, where they found that propofol use was associated with reduced postoperative pain intensity at rest, 30 min, 1 h, and 12 h compared with inhalational anaesthesia (mean difference in pain scores at 30 min, −0.48 [visual analogue scale, 0–10]; 99% CI −1.07 to 0.12, *P*=0.04).

Lastly, our findings suggest an association between the use of sevoflurane for maintenance anaesthesia and an increased likelihood of requiring postoperative rescue analgesia compared with propofol. However, the strength of this association appears moderate, and the 95% CI is fairly wide and includes the null value suggesting some uncertainty about the significance of these results and the true effect size. Moreover, there was moderate heterogeneity among the studies suggesting variability in the results across the included studies. This variability could be the result of differences in study populations, methodologies, or other factors affecting postoperative pain management. Therefore, our results suggest that there is a tendency for patients maintained on sevoflurane to require more rescue analgesia after surgery compared with propofol. These results are comparable with those of the meta-analysis by Zhao and colleagues,^18^ which showed propofol to have an opioid-sparing effect, delaying the first request for rescue analgesia, compared to sevoflurane. Similarly, in the meta-analysis by Peng and colleagues,^44^ it was found that fewer patients required rescue analgesia in the first 24 h postoperatively in the propofol group compared with the inhalational anaesthetic group (risk ratio 0.87, 99% CI 0.74–1.03; *P*=0.04). Moreover, their analysis revealed that patients maintained on propofol required administration of the postoperative analgesic later than those maintained on volatile anaesthetics (mean difference 6.12 min, 99% CI 0.02–12.21; *P*=0.01) and had reduced morphine-equivalent consumption in the first 24 h postoperatively (mean difference −2.68 mg, 99% CI −6.17 to 0.82; *P*=0.05).^44^ This further supports our findings, but cautious interpretation of this outcome is advised because of the heterogeneity and the wide CI. Therefore, further studies are needed to investigate the factors affecting this association, which could provide better insights into the variability in the results of the individual RCTs.

Subgroup analyses suggested consistency in the finding that propofol is associated with less postoperative pain, with a few exceptions. However, subgroup results should always be interpreted with caution as they tend to be chance findings, particularly in subgroups with few RCTs.

There are several possible mechanisms that could potentially explain why propofol may induce less postoperative pain compared with sevoflurane. The first possibility could be that propofol has anti-inflammatory properties, which may contribute to its analgesic effects as suggested by some studies. Propofol has been shown to suppress proinflammatory cytokines and to decrease lipopolysaccharide-induced production of reactive oxygen species.^45^^,^^46^ Furthermore, propofol is thought to induce its anaesthetic impact by amplifying the inhibitory actions of the neurotransmitter gamma-aminobutyric acid (GABA) at the GABA-A receptor. This receptor is extensively distributed in the central nervous system and contributes to pain processing. Through boosting GABA-mediated inhibition, propofol potentially aids in dampening pain signals.^47^ Moreover, it has been suggested that propofol is associated with preventive analgesic effects, which is demonstrated when the drug reduces analgesic use, postoperative pain beyond its duration of action, or both.^48^ One reason for this phenomenon could be propofol's effect on the exchange protein directly activated by 3′–5′-cyclic adenosine monophosphate (EPAC). It has been described to reduce spinal dorsal horn EPAC1 expression in an animal model on postoperative pain.^49^ This is highlighted as EPAC plays a role in causing acute pain to transition to persistent pain.^50^ Another reason could be a result of its inhibitory effect on *N*-methyl-d-aspartate (NMDA) receptors, where inhibition with NMDA antagonists has shown preventive analgesic effects.^51^^,^^52^ In healthy volunteers, propofol has shown transient analgesic effects with pain scores lowered by 38% after acute pain induction, showing diminished hyperalgesia and allodynia.^53^ In animal models, propofol not only suppresses nociception induced by spinal sensitisation but also reduces the responses of lumbar dorsal horn neurones to noxious stimuli.^54^^,^^55^ In contrast, it was reported that inhaled anaesthetics such as sevoflurane, at 0.1 minimum alveolar concentrations, often cause hyperalgesia, potentially contributing to heightened pain perception from anaesthesia.^49^ This increased pain sensitivity is influenced by the modulation of central adrenergic and cholinergic transmission, and by 5-HT3 receptor-mediated currents.^56^^,^^57^

Modulation of hyperpolarisation-activated cyclic nucleotide-gated (HCN) channels is another possible mechanism by which propofol might lessen postoperative pain.^58^^,^^59^ A family of ion channels known as HCN channels is involved in a number of physiological functions, such as heart rate regulation and neuronal excitability.^58^ HCN1–2 subunits, in particular, have been linked to the transmission of electrical signals that trigger the onset of peripheral pain. Consequently, analgesia is produced when these channels are blocked, causing interruption of the signals.^60^ In more detail, HCN channels regulate the electrical excitability of neurons by generating a hyperpolarisation-activated cationic inward current in neurones.^61^ The anaesthetic effects of propofol might result from blocking this inward current in the dorsal root ganglion of central neurones, which are involved in the transmission of pain among other sensory information.^62^

This review has several strengths. This meta-analysis has a good sample size of included RCTs (n=13) and uses a bias-adjusted synthesis method to weigh studies, which has been shown to be more robust than the random effects model when dealing with heterogeneous studies.^25^ However, some limitations remain. Firstly, the quality assessment revealed that some RCTs did not report sufficient details about the randomisation, allocation concealment, or blinding. These poorly reported safeguards resulted in the studies having lower scores in the quality assessment using the MASTER scale.^23^ Secondly, our analysis revealed a moderately high heterogeneity, which could be attributed to the differences in outcome definitions and their measurements.

### Conclusions

This review suggests that children maintained on inhalational sevoflurane anaesthesia had higher odds of having postoperative pain compared with those maintained on propofol anaesthesia. Keeping the aforementioned limitations in mind, better methodological quality RCTs and more studies investigating the relationship between the type of surgery, different intraoperative management, and other associated factors and how they interact with the occurrence of postoperative pain are warranted to provide a clearer answer on the occurrence of postoperative pain when sevoflurane or propofol is used as maintenance anaesthesia in children undergoing surgical procedures.

## Funding

Open access funding provided by the Qatar National Library.

## Authors’ contributions

Study conception and design: BMA, KTS, AS, TC

Acquisition of data: BMA, AME, NET, MA, RFH, AFA, MNH, FRM

Analysis and interpretation of data: BMA, AME, NET, MA, RFH, AFA, TC

Drafting of final manuscript: BMA, AME, NET, MA, RFH, AFA, MNH, FRM

Editing of final manuscript: BMA, AME, KTS, AS, TC

All authors made a significant contribution to the work reported; took part in drafting, revising, or critically reviewing the manuscript; gave final approval of the version to be published; have agreed on the journal to which the manuscript has been submitted; and agree to be accountable for all aspects of the work.

## Availability of data and materials

The data used in this work are available upon reasonable request from the corresponding author.

## Declaration of interest

The authors declare no conflicts of interest in connection with the study reported.
