# Supplementary material for: Comparison of postoperative pain in children after maintenance anaesthesia with propofol or sevoflurane: a systematic review and meta-analysis
Source: Br J Anaesth. 2024 Apr 26;133(1):93–102. doi: 10.1016/j.bja.2024.03.022 (PMC11213989; doi:10.1016/j.bja.2024.03.022)
Supplement: Multimedia component 1 [file mmc1.pdf]

Supplementary Material

Comparison of Postoperative Pain in Children After Maintenance Anaesthesia with Propofol or Sevoflurane: A Systematic Review and Meta-Analysis

Table of Contents

PRISMA Checklist 2

Search Strategy 5

Excluded Reports 8

Assessment of the Quality of Included Studies 10

Supplementary Figures 11

References 17

PRISMA Checklist

Supplementary Table S1. PRISMA Checklist.

Section and Topic	Item #	Checklist item	Location where item is reported
**TITLE**			
Title	1	Identify the report as a systematic review.	1
**ABSTRACT**			
Abstract	2	See the PRISMA 2020 for Abstracts checklist.	2 – 3
**INTRODUCTION**			
Rationale	3	Describe the rationale for the review in the context of existing knowledge.	4 – 5
Objectives	4	Provide an explicit statement of the objective(s) or question(s) the review addresses.	2
**METHODS**			
Eligibility criteria	5	Specify the inclusion and exclusion criteria for the review and how studies were grouped for the syntheses.	6
Information sources	6	Specify all databases, registers, websites, organisations, reference lists and other sources searched or consulted to identify studies. Specify the date when each source was last searched or consulted.	5
Search strategy	7	Present the full search strategies for all databases, registers and websites, including any filters and limits used.	5 – 6
Selection process	8	Specify the methods used to decide whether a study met the inclusion criteria of the review, including how many reviewers screened each record and each report retrieved, whether they worked independently, and if applicable, details of automation tools used in the process.	6
Data collection process	9	Specify the methods used to collect data from reports, including how many reviewers collected data from each report, whether they worked independently, any processes for obtaining or confirming data from study investigators, and if applicable, details of automation tools used in the process.	6 – 7
Data items	10a	List and define all outcomes for which data were sought. Specify whether all results that were compatible with each outcome domain in each study were sought (e.g. for all measures, time points, analyses), and if not, the methods used to decide which results to collect.	6
	10b	List and define all other variables for which data were sought (e.g. participant and intervention characteristics, funding sources). Describe any assumptions made about any missing or unclear information.	6 – 7
Study risk of bias assessment	11	Specify the methods used to assess risk of bias in the included studies, including details of the tool(s) used, how many reviewers assessed each study and whether they worked independently, and if applicable, details of automation tools used in the process.	7
Effect measures	12	Specify for each outcome the effect measure(s) (e.g. risk ratio, mean difference) used in the synthesis or presentation of results.	7 – 8
Synthesis methods	13a	Describe the processes used to decide which studies were eligible for each synthesis (e.g. tabulating the study intervention characteristics and comparing against the planned groups for each synthesis (item #5)).	7 – 8
	13b	Describe any methods required to prepare the data for presentation or synthesis, such as handling of missing summary statistics, or data conversions.	7 – 8

Section and Topic	Item #	Checklist item	Location where item is reported
	13c	Describe any methods used to tabulate or visually display results of individual studies and syntheses.	7 – 8
	13d	Describe any methods used to synthesize results and provide a rationale for the choice(s). If meta-analysis was performed, describe the model(s), method(s) to identify the presence and extent of statistical heterogeneity, and software package(s) used.	7 – 8
	13e	Describe any methods used to explore possible causes of heterogeneity among study results (e.g. subgroup analysis, meta-regression).	7 – 8
	13f	Describe any sensitivity analyses conducted to assess robustness of the synthesized results.	7 – 8
Reporting bias assessment	14	Describe any methods used to assess risk of bias due to missing results in a synthesis (arising from reporting biases).	7 – 8
Certainty assessment	15	Describe any methods used to assess certainty (or confidence) in the body of evidence for an outcome.	7 – 8
**RESULTS**			
Study selection	16a	Describe the results of the search and selection process, from the number of records identified in the search to the number of studies included in the review, ideally using a flow diagram.	8 – 9
	16b	Cite studies that might appear to meet the inclusion criteria, but which were excluded, and explain why they were excluded.	8 – 9
Study characteristics	17	Cite each included study and present its characteristics.	9 – 10
Risk of bias in studies	18	Present assessments of risk of bias for each included study.	10 – 11
Results of individual studies	19	For all outcomes, present, for each study: (a) summary statistics for each group (where appropriate) and (b) an effect estimate and its precision (e.g. confidence/credible interval), ideally using structured tables or plots.	11 – 14
Results of syntheses	20a	For each synthesis, briefly summarise the characteristics and risk of bias among contributing studies.	Supplementary Table S8
	20b	Present results of all statistical syntheses conducted. If meta-analysis was done, present for each the summary estimate and its precision (e.g. confidence/credible interval) and measures of statistical heterogeneity. If comparing groups, describe the direction of the effect.	11 – 14
	20c	Present results of all investigations of possible causes of heterogeneity among study results.	11 – 14
	20d	Present results of all sensitivity analyses conducted to assess the robustness of the synthesized results.	11 – 14
Reporting biases	21	Present assessments of risk of bias due to missing results (arising from reporting biases) for each synthesis assessed.	11 – 14
Certainty of evidence	22	Present assessments of certainty (or confidence) in the body of evidence for each outcome assessed.	15
**DISCUSSION**			
Discussion	23a	Provide a general interpretation of the results in the context of other evidence.	15 – 18
	23b	Discuss any limitations of the evidence included in the review.	18
	23c	Discuss any limitations of the review processes used.	18

Section and Topic	Item #	Checklist item	Location where item is reported
	23d	Discuss implications of the results for practice, policy, and future research.	18
**OTHER INFORMATION**			
Registration and protocol	24a	Provide registration information for the review, including register name and registration number, or state that the review was not registered.	5
	24b	Indicate where the review protocol can be accessed, or state that a protocol was not prepared.	5
	24c	Describe and explain any amendments to information provided at registration or in the protocol.	Not applicable
Support	25	Describe sources of financial or non-financial support for the review, and the role of the funders or sponsors in the review.	19
Competing interests	26	Declare any competing interests of review authors.	19
Availability of data, code and other materials	27	Report which of the following are publicly available and where they can be found: template data collection forms; data extracted from included studies; data used for all analyses; analytic code; any other materials used in the review.	19

Search Strategy

Supplementary Table S2. PubMed Medline Search. Results: 330. Last updated: 15 July 2023.

("pain, postoperative"[MeSH Terms] OR "postoperative pain"[All Fields] OR "post operative pain"[All Fields] OR “rescue analges*” [All Fields] OR ("rescue"[All Fields] AND "analgesi*"[All Fields]) OR (("postoperative period"[MeSH Terms] OR ("postoperative"[All Fields] AND "period"[All Fields]) OR "postoperative period"[All Fields] OR "postop"[All Fields] OR "postoperative"[All Fields] OR "postoperatively"[All Fields]) AND "analgesi*"[All Fields])) <br>AND <br>("Propofol"[MeSH Terms] OR "propofo*"[All Fields] OR ("intraven*"[All Fields] AND "anesthe*"[All Fields]) OR "TIVA"[All Fields]) <br>AND <br>("Sevoflurane"[MeSH Terms] OR "Sevoflurane"[All Fields] OR "sevoflu*"[All Fields] OR "sevo"[All Fields] OR (("administration, inhalation"[MeSH Terms] OR "inhalation"[MeSH Terms] OR "inhalation"[All Fields]) AND "anesthe*"[All Fields])) <br>AND <br>("child"[tiab] OR "children"[tiab] OR "child*"[tiab] OR "infant*"[tiab] OR "teenage*"[tiab] OR "adolescent*"[tiab])

Supplementary Table S3. Embase Search. Results: 162. Last updated: 15 July 2023.

('postoperative pain:ti,ab' OR 'post operative pain:ti,ab' OR 'rescue analges*:ti,ab' OR (rescue AND analgesi*:ti,ab) OR (((postoperative AND period) OR 'postoperative period' OR postop OR postoperative OR postoperatively) AND analgesi*:ti,ab)) <br>AND <br>(propofo*:ti,ab OR (intraven* AND anesthe*:ti,ab) OR TIVA:ti,ab) <br>AND <br>(Sevoflurane:ti,ab OR sevoflu*:ti,ab OR sevo:ti,ab OR (inhalation AND anesthe*:ti,ab)) <br>AND <br>(child:ti,ab OR children:ti,ab OR child*:ti,ab OR infant*:ti,ab OR teenage*:ti,ab OR adolescent*:ti,ab)

Supplementary Table S4. Scopus Search. Results: 1049. Last updated: 15 July 2023.

(TITLE-ABS-KEY("postoperative pain") OR TITLE-ABS-KEY("post operative pain") OR TITLE-ABS-KEY("rescue* analges*") OR (TITLE-ABS-KEY(rescue) AND TITLE-ABS-KEY(analgesi*)) OR (((TITLE-ABS-KEY(postoperative) AND TITLE-ABS-KEY(period)) OR TITLE-ABS-KEY("postoperative period") OR TITLE-ABS-KEY(postop) OR TITLE-ABS-KEY(postoperative) OR TITLE-ABS-KEY(postoperatively)) AND TITLE-ABS-KEY(analgesi*)))
AND
(TITLE-ABS-KEY(propofo*)) OR (TITLE-ABS-KEY(intraven*) AND TITLE-ABS-KEY(anesthe*)) OR (TITLE-ABS-KEY(TIVA))
AND
(TITLE-ABS-KEY(Sevoflurane)) OR (TITLE-ABS-KEY(sevoflu*)) OR (TITLE-ABS-KEY(sevo)) OR (TITLE-ABS-KEY(inhalation) AND TITLE-ABS-KEY(anesthe*))
AND
(TITLE-ABS-KEY(child)) OR (TITLE-ABS-KEY(children)) OR (TITLE-ABS-KEY(child*)) OR (TITLE-ABS-KEY(infant*)) OR (TITLE-ABS-KEY(teenage*)) OR (TITLE-ABS-KEY(adolescent*))

Supplementary Table S5. Cochrane Library Search. Results: 195, including 2 trials and 193 reviews. Last updated: 16 July 2023.

("postoperative pain":ti,ab OR "post operative pain":ti,ab OR ("rescue" NEXT analges*):ti,ab OR (rescue:ti,ab AND analgesi*:ti,ab) OR (((postoperative:ti,ab AND period:ti,ab) OR "postoperative period":ti,ab OR postop:ti,ab OR postoperative:ti,ab OR postoperatively:ti,ab) AND analgesi*:ti,ab))
AND
(propofo*:ti,ab OR (intraven*:ti,ab AND anesthe*:ti,ab) OR TIVA:ti,ab)
AND
(Sevoflurane:ti,ab OR sevoflu*:ti,ab OR sevo:ti,ab OR (inhalation:ti,ab AND anesthe*:ti,ab))
AND
(child:ti,ab OR children:ti,ab OR child*:ti,ab OR infant*:ti,ab OR teenage*:ti,ab OR adolescent*:ti,ab)

Supplementary Table S6. Web of Science Search. Results: 74. Last updated: 16 July 2023.

((TI="postoperative pain" OR AB="postoperative pain") OR (TI="post operative pain" OR AB="post operative pain") OR (TI="rescue analges*" OR AB="rescue analges*") OR ((TI=rescue OR AB=rescue) AND (TI=analgesi* OR AB=analgesi*)) OR ((((TI=postoperative OR AB=postoperative) AND (TI=period OR AB=period)) OR (TI="postoperative period" OR AB="postoperative period") OR (TI=postop OR AB=postop) OR (TI=postoperative OR AB=postoperative) OR (TI=postoperatively OR AB=postoperatively)) AND (TI=analgesi* OR AB=analgesi*)))
AND
((TI=propofo* OR AB=propofo*) OR ((TI=intraven* OR AB=intraven*) AND (TI=anesthe* OR AB=anesthe*)) OR (TI=TIVA OR AB=TIVA))
AND
((TI=Sevoflurane OR AB=Sevoflurane) OR (TI=sevoflu* OR AB=sevoflu*) OR (TI=sevo OR AB=sevo) OR ((TI=inhalation OR AB=inhalation) AND (TI=anesthe* OR AB=anesthe*)))
AND
((TI=child OR AB=child) OR (TI=children OR AB=children) OR (TI=child* OR AB=child*) OR (TI=infant* OR AB=infant*) OR (TI=teenage* OR AB=teenage*) OR (TI=adolescent* OR AB=adolescent*))

Excluded Reports

Supplementary Table S7. Excluded reports at full-text screening stage with the reasons for exclusion.

First Author	Year	Title	Reason for Exclusion
Hobbhahn, J. (1)	1997	Induction, emergence and recovery with sevofluran and desfluran in comparison to other inhalation anaesthetics and propofol.	Wrong study design
Baykara, N. (2)	1998	Comparison of propofol and sevoflurane anaesthesia regarding recovery time and vomiting incidence in outpatient surgery.	Full-text not retrieved
Oddby, E. (3)	1998	Postoperative nausea and vomiting (PONV) in pediatric outpatients: sevoflurane vs. spinal anesthesia with propofol sedation.	Full-text not retrieved
Picard, V. (4)	2000	Quality of recovery in children: sevoflurane versus propofol.	Wrong outcome
Kubo, S. (5)	2001	Recovery characteristics of propofol anesthesia in pediatric outpatients; comparison with sevoflurane anesthesia.	Full-text not retrieved
Durlu, N. (6)	2003	The Effects of Propofol and Sevoflurane Anesthesia on Postoperative Vomiting and Pain in Children Undergoing Strabismus Surgery.	Full-text not retrieved
Gouda, N. (7)	2003	Clinical comparison of single agent anaesthesia with sevoflurane versus total intravenous anaesthesia with propofol in preschool children undergoing day case surgery.	Full-text not retrieved
Hanna, M.G. (8)	2004	Comparison of two rapid recovery anaesthetic techniques using propofol versus sevoflurane in adjustable strabismus surgery.	Full-text not retrieved
Sarti, A. (9)	2004	Incidence of vomiting in susceptible children under regional analgesia with two different anaesthetic techniques.	POP not measured adequately
Šimurina, T. (10)	2006	The effect of propofol and fentanyl as compared with sevoflurane on postoperative vomiting in children after adenotonsillectomy.	Wrong outcome
Nakayama, S. (11)	2007	Propofol reduces the incidence of emergence agitation in preschool-aged children as well as in school-aged children: a comparison with sevoflurane.	Wrong outcome
Steinmetz, J. (12)	2007	Hemodynamic differences between propofol-remifentanil and sevoflurane anesthesia for repair of cleft lip and palate in infants.	POP not measured adequately
Chung, J.H. (13)	2010	Vomiting after a pediatric adenotonsillectomy: comparison between propofol induced	Wrong outcome

		sevoflurane-nitrous oxide maintained anesthesia and TIVA with propofol-remifentanil.	
Pieters, B. J. (14)	2010	Emergence delirium and postoperative pain in children undergoing adenotonsillectomy: a comparison of propofol vs sevoflurane anesthesia.	POP not measured adequately
Mahoney, E. (15)	2011	Local Anaesthetic Use in Dental General Anaesthetics on Paediatric Patients undergoing tooth extraction and their reported post operative pain.	Full-text not retrieved (Report not published)
Myers, D. (16)	2012	TIVA reduces emergence delirium in children.	Wrong publication type
Song, J.G. (17)	2012	Incidence of post-thoracotomy pain: a comparison between total intravenous anaesthesia and inhalation anaesthesia.	Wrong population
Kocaturk, O. (18)	2018	Recovery characteristics of total intravenous anesthesia with propofol versus sevoflurane anesthesia: a prospective randomized clinical trial.	POP not measured adequately
Atef, H. M. (19)	2019	Postoperative analgesia in children undergoing adenotonsillectomy under sevoflurane versus propofol-based anesthesia: a randomized controlled trial.	POP not measured adequately
Fathy, S. (20)	2020	Propofol Versus Sevoflurane Anesthesia in Pediatric Strabismus Surgery: Feasibility of BIS Monitoring.	Full-text not retrieved (Report not published)
Wang, Y. (21)	2021	A comparative study of the analgesic effects of sevoflurane and propofol in children following otolaryngology surgical procedures: A pilot study.	POP not measured adequately
Khanna, P. (22)	2022	Use of fNIRS to compare Emergence Delirium in children undergoing Sevoflurane Vs Propofol anaesthesia.	Full-text not retrieved (Report not published)

Abbreviations: POP, Postoperative pain.

Assessment of the Quality of Included Studies

Supplementary Table S8. Scores for the assessment of the quality of included studies using the MASTER Scale.

Study	Equal recruitment				Equal retention					Equal ascertainment							Equal implementation						Equal prognosis						Sufficient analysis			Temporal precedence					Total
	1	2	3	4	5	6	7	8	9	10	11	12	13	14	15	16	17	18	19	20	21	22	23	24	25	26	27	28	29	30	31	32	33	34	35	36	
Guard 1998	1	1	1	1	1	1	1	1	1	1	1	1	1	1	0	0	1	1	1	1	1	1	1	1	1	1	1	0	1	1	1	1	1	1	1	0	32
Rusch 1999	1	1	1	1	1	1	1	1	1	1	1	1	1	1	0	0	1	1	1	1	1	1	1	1	1	0	0	0	1	1	1	1	1	1	1	1	31
Lovstad 2001	1	1	1	1	1	1	1	1	1	1	1	1	1	1	0	0	1	1	1	1	1	1	1	1	1	1	1	0	1	1	1	1	1	1	1	1	33
Schmidt 2001	1	1	1	1	1	1	1	1	1	1	1	1	1	0	0	0	1	1	1	1	1	1	1	1	1	0	0	0	1	1	1	1	1	1	1	0	29
Cohen 2003	1	1	1	1	1	1	1	1	1	1	1	1	1	1	0	0	1	1	1	1	1	1	1	1	1	0	0	0	1	1	1	1	1	1	1	0	30
Cohen 2004	1	1	1	1	1	1	1	1	1	1	1	1	1	0	0	0	1	1	1	1	1	1	1	1	1	0	0	0	1	1	1	1	1	1	1	0	29
Auerswald 2006	1	1	1	1	1	1	1	1	1	1	1	1	0	0	0	0	1	0	1	1	1	1	1	1	1	0	0	1	1	1	1	1	1	1	1	0	28
Chandler 2013	1	1	1	1	1	1	1	1	1	1	1	1	1	1	0	0	1	1	1	1	1	1	1	1	1	1	1	1	1	1	1	1	1	1	1	0	33
Hasani 2013	0	1	1	1	1	1	1	1	0	1	1	1	1	1	0	0	1	1	1	1	1	1	1	1	1	1	0	1	1	1	1	1	1	1	1	0	30
Oriby 2021	1	1	1	1	1	1	1	1	1	1	1	1	1	1	0	0	1	1	1	1	1	1	1	1	1	1	0	1	1	1	1	1	1	1	1	0	32
Sheikhzade 2021	1	1	1	1	1	1	1	1	1	1	1	1	0	0	0	0	1	1	1	1	1	1	1	1	1	1	0	1	1	1	1	1	1	1	1	0	30
Lopez 1999	1	1	1	1	1	1	1	1	1	1	1	1	1	0	0	0	1	1	1	1	1	1	1	1	1	0	1	0	1	1	1	1	1	1	1	0	30
Konig 2009	1	1	1	1	1	1	1	1	1	1	1	1	1	0	0	0	1	1	1	1	1	1	1	1	1	1	0	1	1	1	1	1	1	1	1	0	31

Supplementary Figures

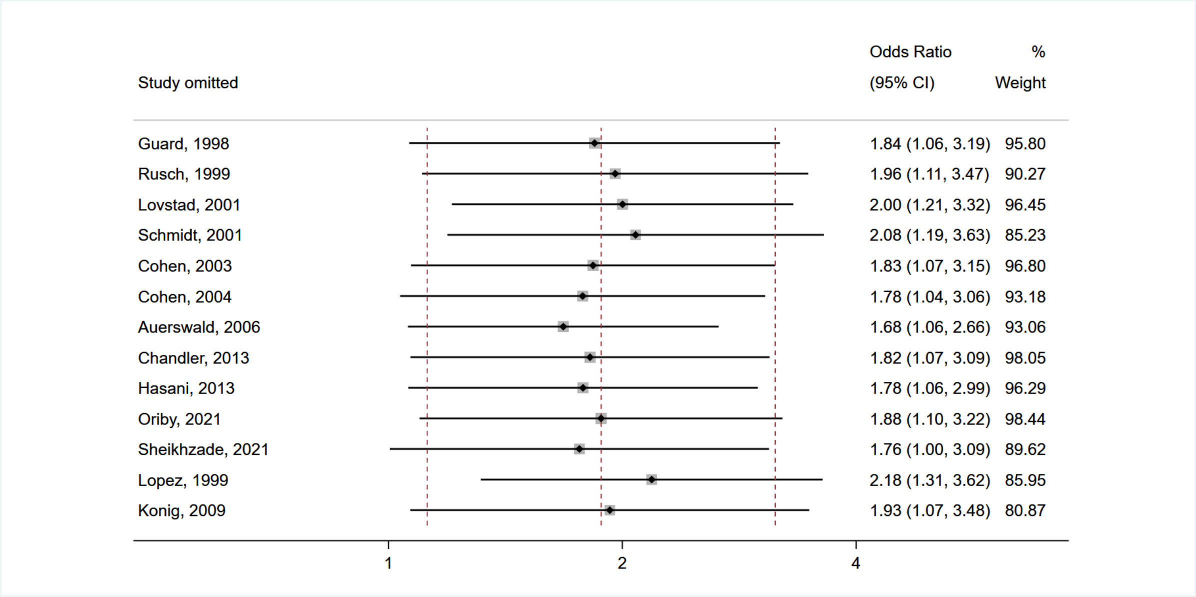

Supplementary Figure S1. Leave-one-out analysis for the primary outcome (overall postoperative pain).

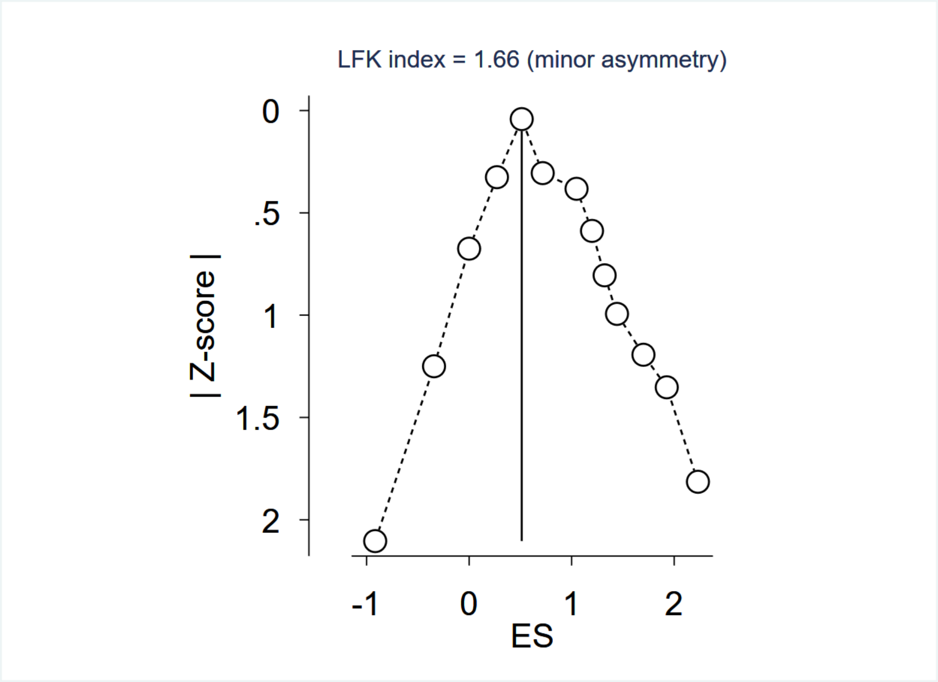

Supplementary Figure S2. Doi Plot and LFK index for the primary outcome (overall postoperative pain).

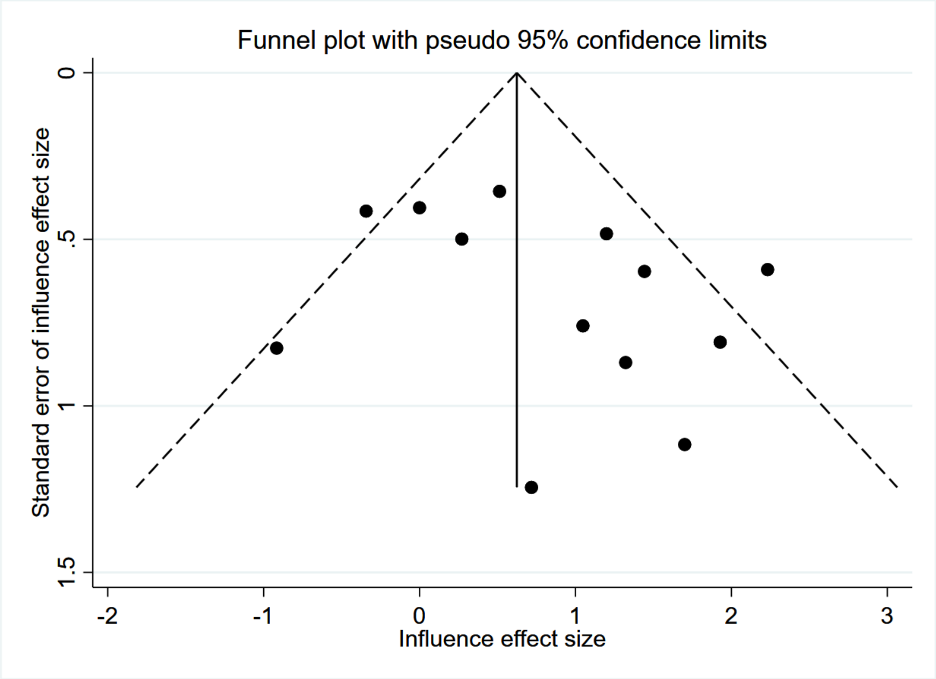

Supplementary Figure S3. Funnel Plot for the primary outcome (overall postoperative pain).

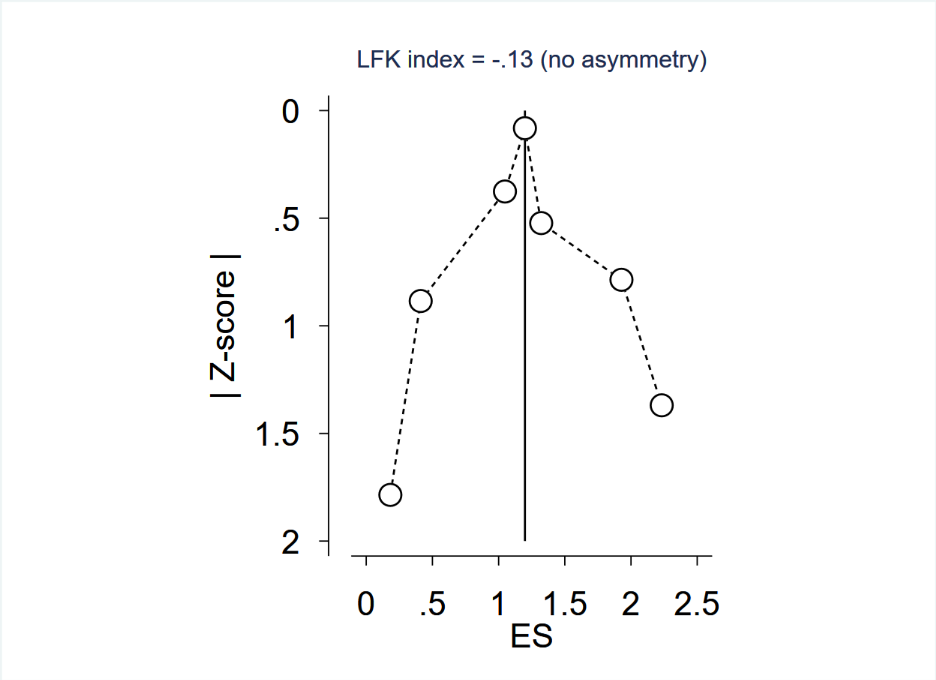

Supplementary Figure S4. Doi Plot and LFK index for the secondary outcome (postoperative pain measured using pain assessment tools only).

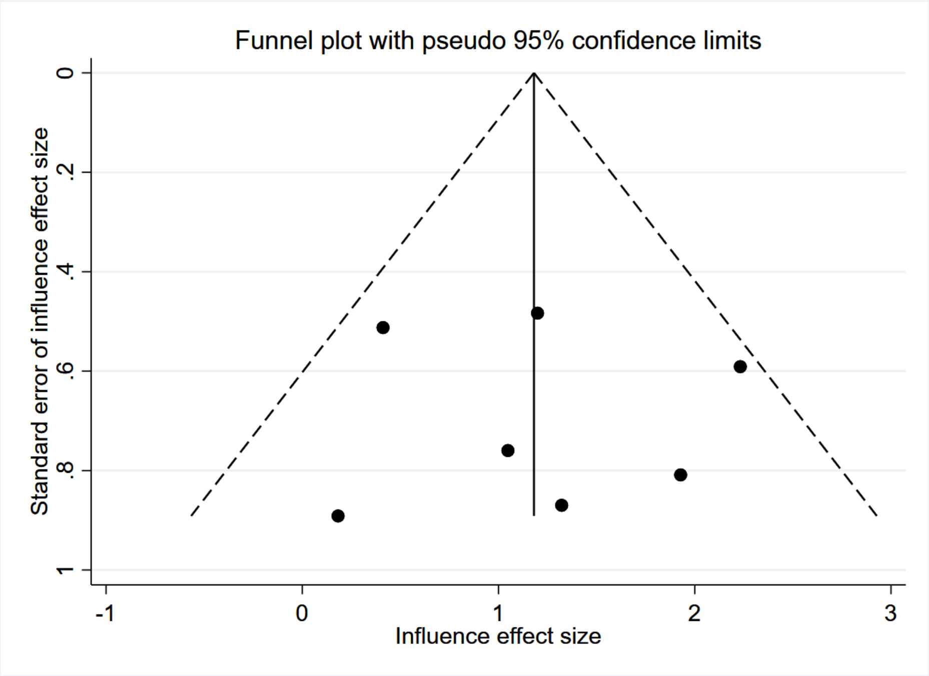

Supplementary Figure S5. Funnel Plot for the secondary outcome (postoperative pain measured using pain assessment tools only).

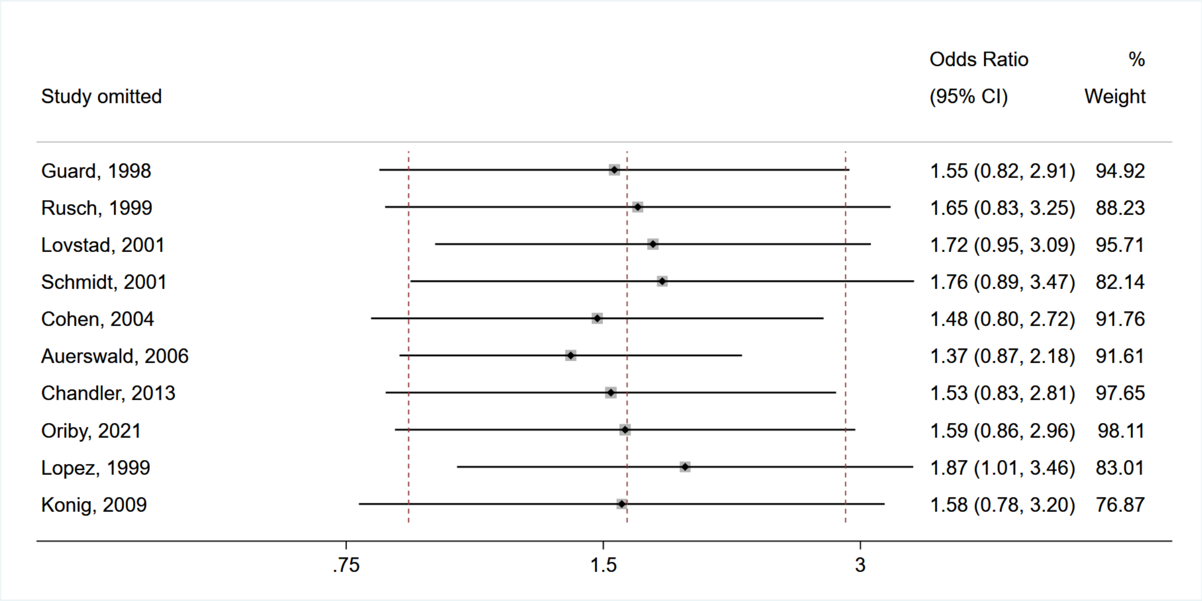

Supplementary Figure S6. Leave-one-out analysis for the secondary outcome (requirement of rescue analgesia).

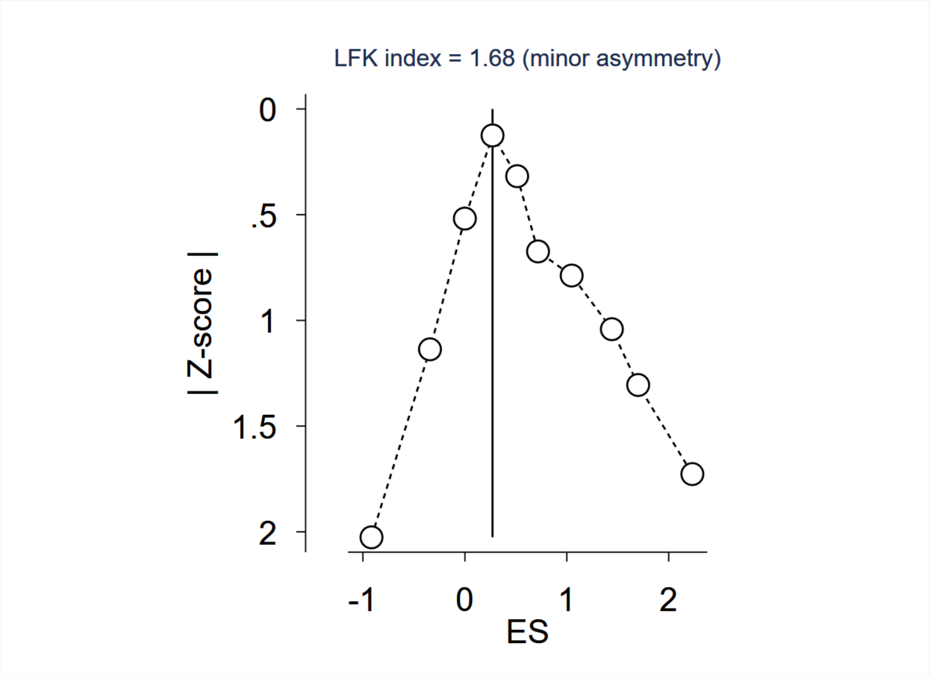

Supplementary Figure S7. Doi Plot and LFK index for the secondary outcome (requirement of rescue analgesia).

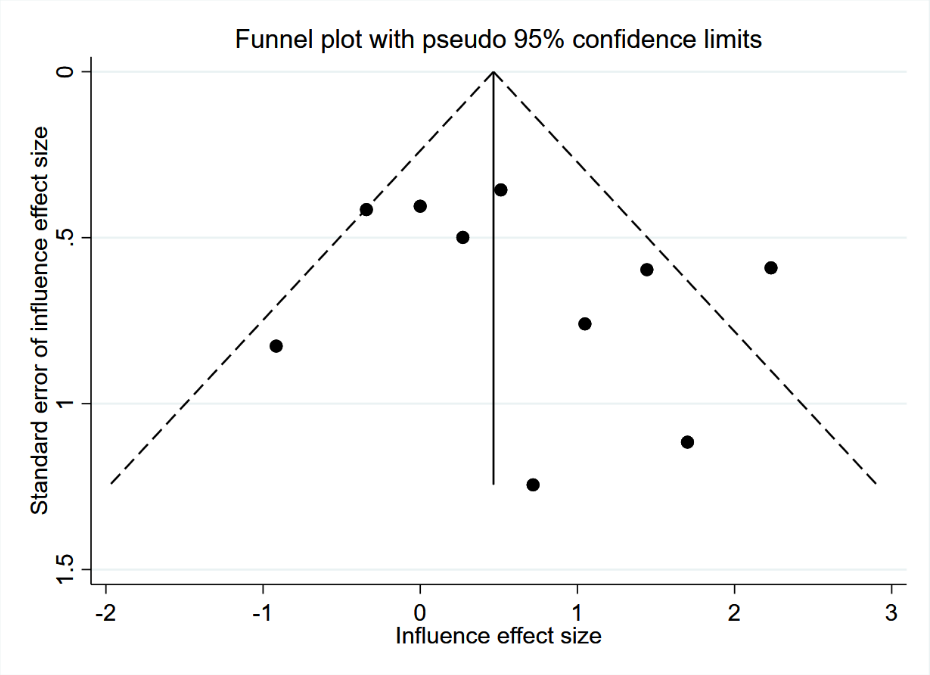

Supplementary Figure S8. Funnel Plot for the secondary outcome (requirement of rescue analgesia).

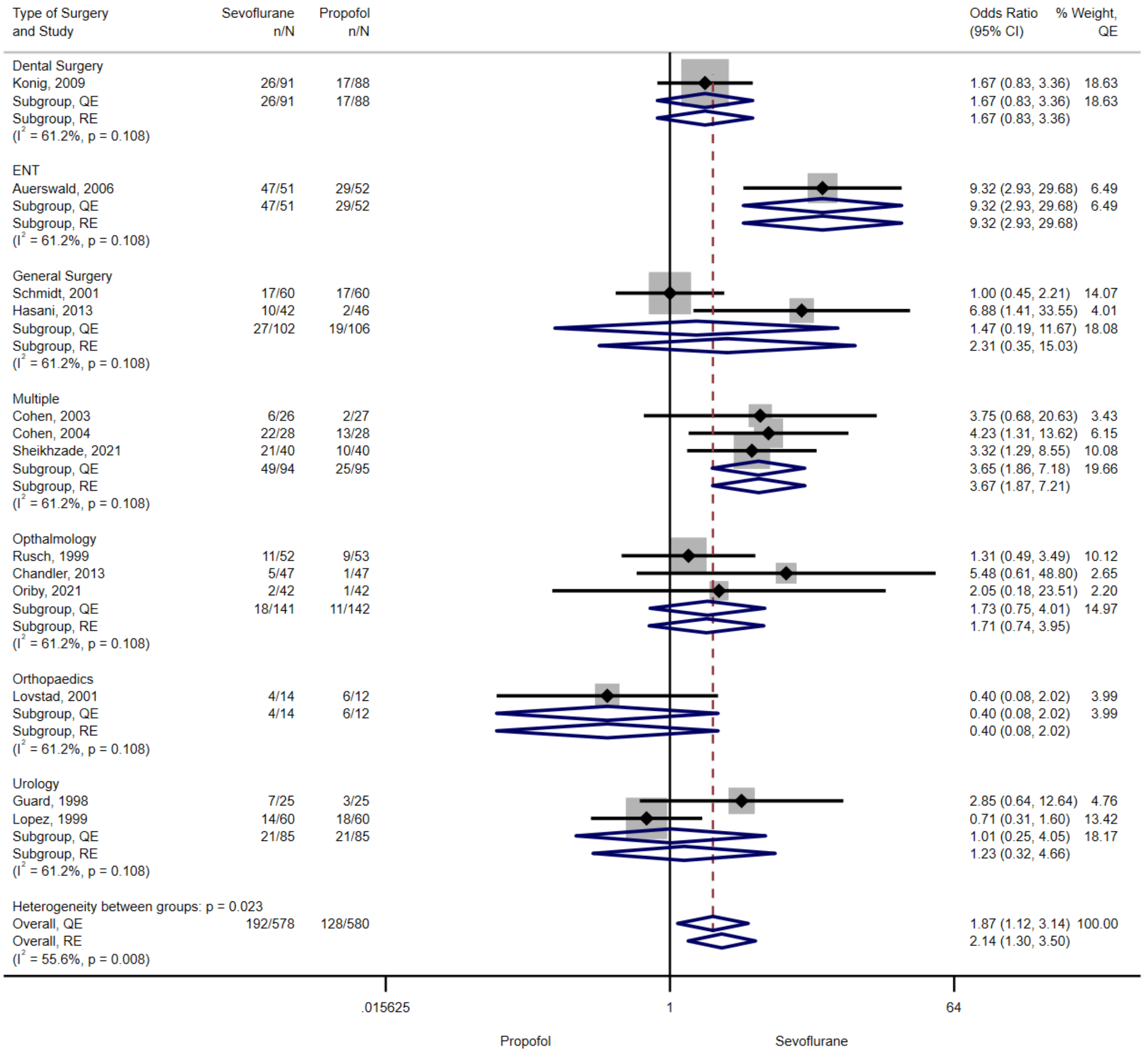

Supplementary Figure S9. Forest Plot for the subgroup analysis by type of surgery.

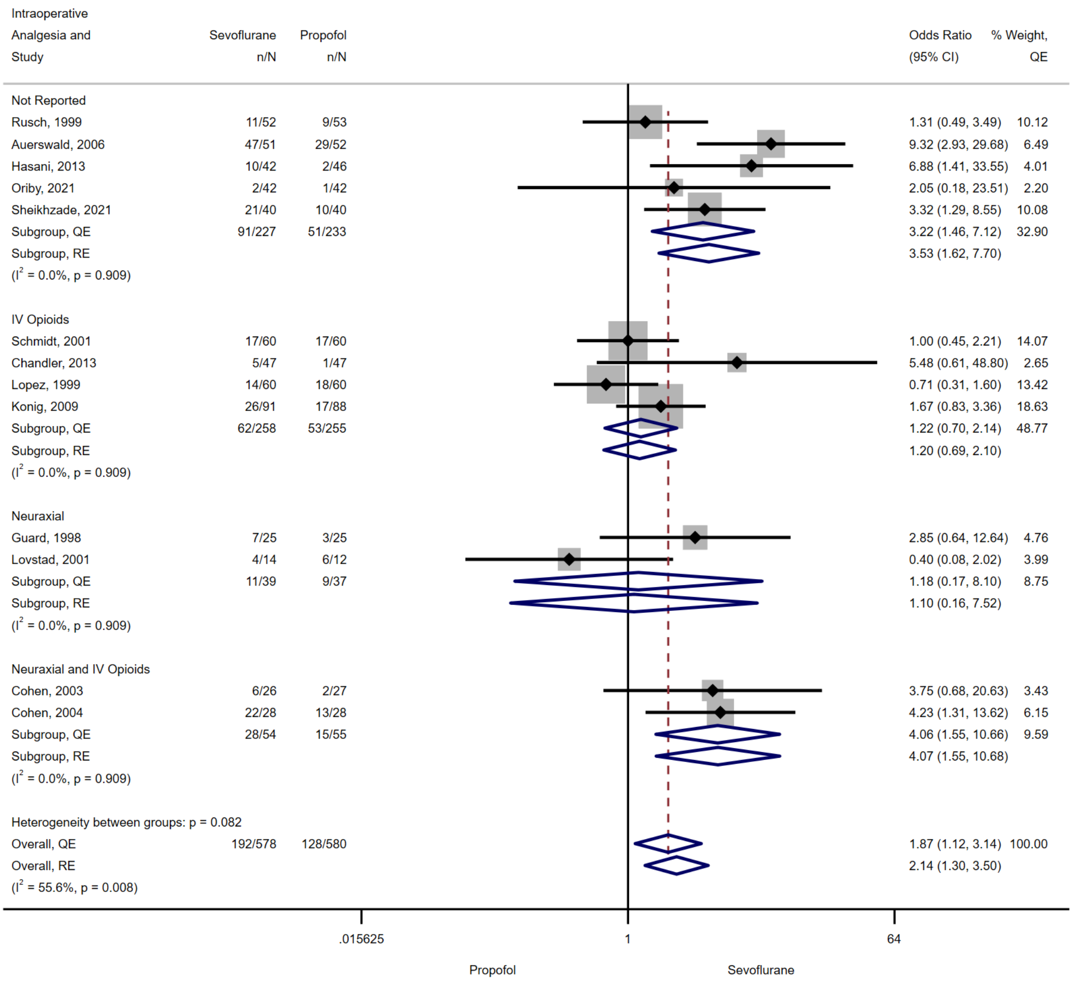

Supplementary Figure S10. Forest Plot for the subgroup analysis by intraoperative analgesic management.

References

1. Hobbhahn J, Schwall B, Prasser C, Vogel H, Taeger K. Induction, emergence and recovery with sevofluran and desfluran in comparison to other inhalation anaesthetics and propofol. Anasthesiologie und Intensivmedizin. 1997;38(12):607-15.
2. Baykara N, Kilickan LT, Indelen S, Karabey F, Toker K. Comparison of propofol and sevoflurane anaesthesia regarding recovery time and vomiting incidence in outpatient surgery. Turk Anesteziyoloji ve Reanimasyon. 1998;26(8):387-91.
3. Oddby E, Reinhard J, Hirsch K, Englund S, Lönnqvist PA. Postoperative nausea and vomiting (PONV) in pediatric outpatients: sevoflurane vs. spinal anesthesia with propofol sedation. Regional Anesthesia & Pain Medicine. 1998;23(Suppl 1):15-.
4. Picard V, Dumont L, Pellegrini M. Quality of recovery in children: sevoflurane versus propofol. Acta Anaesthesiologica Scandinavica. 2000;44(3):307-10.
5. Kubo S, Kinouchi K, Taniguchi A, Fukumitsu K, Kitamura S. [Recovery characteristics of propofol anesthesia in pediatric outpatients; comparison with sevoflurane anesthesia]. Masui. 2001;50(4):371-7.
6. Durlu N, Özlü O. The effects of propofol and sevoflurane anesthesia of postoperative vomiting and pain in children undergoing strabismus surgery. Turk Anesteziyoloji ve Reanimasyon. 2003;31(3):144-9.
7. Gouda N, El Gohary M, Raouf A. Clinical comparison of single agent anaesthesia with sevoflurane versus total intravenous anaesthesia with propofol in preschool children undergoing day case surgery. Egyptian Journal of Anaesthesia. 2003;19(1):59-66.
8. Hanna MG, Said HA. Comparison of two rapid recovery anaesthetic techniques using propofol versus sevoflurane in adjustable strabismus surgery. Egyptian Journal of Anaesthesia. 2004;20(1):59-62.
9. Sarti A, Busoni P, Dell'Oste C, Bussolin L. Incidence of vomiting in susceptible children under regional analgesia with two different anaesthetic techniques. Pediatric Anesthesia. 2004;14(3):251-5.
10. Šimurina T, Mikulandra S, Mraović B, Sonicki Z, Kovačić M, Dželalija B, et al. The effect of propofol and fentanyl as compared with sevoflurane on postoperative vomiting in children after adenotonsillectomy. Collegium antropologicum. 2006;30(2):343-7.

11. Nakayama S, Furukawa H, Yanai H. Propofol reduces the incidence of emergence agitation in preschool-aged children as well as in school-aged children: A comparison with sevoflurane. Journal of Anesthesia. 2007;21(1):19-23.

12. Steinmetz J, Holm-Knudsen R, Sorensen MK, Eriksen K, Rasmussen LS. Hemodynamic differences between propofol-remifentanil and sevoflurane anesthesia for repair of cleft lip and palate in infants. Pediatric Anesthesia. 2007;17(1):32-7.

13. Chung JH, Kim YH, Ko YK, Lee SY, Nam YT, Yoon SH. Vomiting after a pediatric adenotonsillectomy: Comparison between propofol induced sevoflurane-nitrous oxide maintained anesthesia and TIVA with propofol-remifentanil. Korean Journal of Anesthesiology. 2010;59(3):185-9.

14. Pieters BJ, Penn E, Nicklaus P, Bruegger D, Mehta B, Weatherly R. Emergence delirium and postoperative pain in children undergoing adenotonsillectomy: a comparison of propofol vs sevoflurane anesthesia. Pediatric Anesthesia. 2010;20(10):944-50.

15. Mahoney E. Local Anaesthetic Use in Dental General Anaesthetics on Paediatric Patients undergoing tooth extraction. http://wwwwhoint/trialsearch/Trial2aspx?TrialID=ACTRN12611000985987. 2011.

16. Myers D, Chandler JR, Mehta D, Whyte E, Misse MK, Ansermino JM, et al. TIVA reduces emergence delirium in children. Canadian Journal of Anesthesia. 2012;59.

17. Song JG, Shin JW, Lee EH, Choi DK, Bang JY, Chin JH, et al. Incidence of post-thoracotomy pain: A comparison between total intravenous anaesthesia and inhalation anaesthesia. European Journal of Cardio-thoracic Surgery. 2012;41(5):1078-82.

18. Kocaturk O, Keles S. Recovery characteristics of total intravenous anesthesia with propofol versus sevoflurane anesthesia: a prospective randomized clinical trial. Journal of Pain Research. 2018;11:1289-95.

19. Atef HM, Ismail SA, Al-Touny A, Abo-rehab SEA. Postoperative analgesia in children undergoing adenotonsillectomy under sevoflurane versus propofol-based anesthesia: a randomized controlled trial. Ain-Shams Journal of Anesthesiology. 2019;11(1):1-10.

20. Fathy S. Propofol Versus Sevoflurane Anesthesia in Pediatric Strabismus Surgery: feasibility of BIS Monitoring. https://clinicaltrialsgov/show/NCT04485117. 2020.

21. Wang Y, Yao F, Lin Y, Xiao S. A comparative study of the analgesic effects of sevoflurane and propofol in children following otolaryngology surgical procedures: A pilot study. Tropical Journal of Pharmaceutical Research. 2021;20(1):211-7.

22. Khanna P. Use of fNIRS to compare Emergence Delirium in children undergoing Sevoflurane Vs Propofol anaesthesia. https://trialsearchwhoint/Trial2aspx?TrialID=CTRI/2022/04/041573. 2022.
